# Increased risk of thromboembolism in patients with malignant lymphoma: a single-centre analysis

**DOI:** 10.1038/sj.bjc.6602504

**Published:** 2005-03-29

**Authors:** M Mohren, I Markmann, K Jentsch-Ullrich, M Koenigsmann, G Lutze, A Franke

**Affiliations:** 1 Klinik für Hämatologie/Onkologie, Universität Magdeburg, Leipziger Str. 44, 39120 Magdeburg, Germany; 2 Institut für Klinische Chemie, Universität Magdeburg, Leipziger Str. 44, 39120 Magdeburg, Germany

**Keywords:** thromboembolism, risk, malignant lymphoma

## Abstract

An increased risk for thromboembolism in cancer patients has been observed in patients with solid tumours, whereas little data exist on malignant lymphoma. We found an overall thromboembolic event incidence of 7.7% in 1038 lymphoma patients treated in our institution, with a statistically significantly higher incidence in high-grade than in low-grade lymphoma.

A thromboembolic event (TE) occurs in approximately 15% of patients with malignant disease (Deitcher, 2003) and cancer patients who develop thromboembolism usually have shorter survival (Sorensen *et al*, 2000). Additionally, cancer is frequently found in patients admitted for idiopathic TE (odds ratio 3.2) or develops subsequently (Baron *et al*, 1998; Murchison *et al*, 2004). Cancers of the lung, brain, liver and kidney seem to carry a particularly high risk for TE (Baron *et al*, 1998), additional risk factors being extensive malignant disease as well as chemo- and/or hormonal therapy (Prandoni *et al*, 1992; Sorensen *et al*, 1998; Lee and Levine, 2003). Cancer patients with TE have a higher fatality and recurrence rate than patients with idiopathic TE (Cushman *et al*, 2004). There exist little data on the incidence of TE in patients with haematologic malignancies: three earlier studies suggested an increased incidence of thrombosis in patients with high-grade non-Hodgkin's lymphoma (hgNHL) (Ottinger *et al*, 1995), Hodgkin's disease (HD) (Seifter *et al*, 1985) and CNS lymphoma (Goldschmidt *et al*, 2003). To our knowledge, there exist no data on the TE incidence in patients with low-grade non-Hodgkin's lymphoma (lgNHL) so far. Large cancer registry studies also showed an increased risk of NHL and HD in patients within a year after a venous TE (Baron *et al*, 1998; Murchison *et al*, 2004). However, patient numbers were either small or no detailed information on the lymphoma subtype was provided, and analysis rather looked at cancer risk in a thrombosis patient than at thrombosis risk in lymphoma patients. Therefore, we initiated a retrospective single-centre investigation on thromboembolism in patients with malignant lymphoma.

## PATIENTS AND METHODS

We reviewed all medical records of patients with malignant lymphoma treated at our institution between January 1991 and July 2004. Patients with HD and NHL according to the Kiel/WHO classification were included. Patients with multiple myeloma, benign lymphadenopathy or acute lymphoblastic leukaemia were excluded from analysis. Thromboembolic events had been confirmed by venography or ultrasound (deep venous thrombosis (DVT), upper extremity thrombosis, central venous catheter thrombosis, portal vein thrombosis), computed tomography or szintigraphy (pulmonary embolism (PE)), MRI (central nervous system (CNS) thrombosis) or angiogram (arterial thrombosis). In lymphoma patients with TE, medical records were thoroughly reviewed for additional thrombophilic risk factors such as comorbid solid tumours, and, if available, results of laboratory work-up for thrombophilia.

Lymphoma patients were catagorised into four distinct clinical groups: hgNHL, lgNHL (distinction according to the Kiel classification), HD and primary CNS lymphoma. For easier analysis, patients with high-grade gastric lymphoma were staged according to the Ann Arbor staging system, for example, stage I_E_A if no further lymphadenopathy was found. Data were collected and analysed in a Microsoft Excel database.

### Statistical analysis

*P*-values to show correlation of the TE rate with histologic subtype, gender, age and disease stage were calculated using Fisher's exact test. All reported *P*-values are two-sided. Due to the small sample size, patients with CNS lymphoma were omitted from the histologic subtype correlation analysis.

## RESULTS

A total of 1038 patients, with malignant lymphoma were eligible for analysis: 569 patients were men (54.8%) and 469 were women (45.2%), and mean age was 59.2 years. A total of 348 patients had high-grade lymphoma, 485 had low-grade lymphoma, 193 had HD and 12 had primary CNS lymphoma. Out of 1038 lymphoma patients, 80 (7.7%) had at least one TE. A total of 96 TE occurred in this patient group. A total of 38 patients were men and 42 women. Thromboembolic events included DVT (*n*=51), PE (*n*=19), central venous catheter thrombosis (*n*=11), upper extremity thrombosis due to tumour compression (*n*=9), CNS thrombosis (*n*=3), arterial thrombosis (*n*=2) and portal vein thrombosis (*n*=1). Patients with central venous catheter thrombosis had high-grade lymphoma (*n*=4), low-grade lymphoma (*n*=3) and HD (*n*=4). A total of 69 TE (72%) occurred during treatment, whereas 27 (28%) were diagnosed prior to (*n*=16) (17%) or after completion (*n*=11) (11%) of therapy.

Patients with hgNHL had a higher rate of TE (10.6%) compared to patients with HD (7.25%) and lgNHL (5.8%), although the difference was statistically significant in comparison with lgNHL only (Tables 1 and 2). A statistically significant difference between high-grade and low-grade lymphoma was maintained even if patients with central venous catheter thrombosis were omitted from analysis (*P*=0.018). Subgroup analysis of patients with high grade lymphoma showed a slightly higher TE rate in female patients (12.6 *vs* 8.7%) (Table 3) and in patients with advanced disease (stage III and IV) (12.6 *vs* 8.6%) (Table 5) whereas no difference was found for age below or above 60 years (Table 4). In contrast to these findings, patients with HD at 60 years or older had a higher rate of TE compared to patients younger than 60 years (13.3 *vs* 5.4%) (Table 4), but no difference regarding gender or disease stage (Tables 3 and 5) was found. However, all these findings failed to reach statistical significance due to the overall low number of thromboembolic events.

## DISCUSSION

Our analysis shows an increased incidence of TE of approximately 8% in patients with malignant lymphoma. The highest incidence was observed among patients with hgNHL, whereas a much lower incidence was seen in patients with lgNHL.

A 6.6% incidence of venous thromboembolism was found in a prospective study of 593 patients with hgNHL, the majority (80%) of which occurred within the first 3 months of therapy. Thromboembolic event was associated with advanced stage and an unfavourable clinical course (Ottinger *et al*, 2004). Retrospective analysis of 177 patients with HD revealed 6% DVT occurring in the absence of detectable tumour (Seifter *et al*, 1987), and another study showed a high incidence of TE in patients with CNS lymphoma (Goldschmidt *et al*, 2003). Our results are in concordance with these findings, showing a slightly higher TE rate in patients with hgNHL than in the study by Ottinger *et al*, possibly due to the fact that patients with DVT only were included. To our knowledge there exist no data on the TE rate in patients with low-grade lymphoma so far. In this cohort, correlation analysis of TE rate and disease stage was omitted for two reasons: (1) the overall TE rate in this patient group was rather low and (2) a large portion of the patients with low-grade lymphoma had CLL (187/485=38.5%), a disease with a staging system different from the Ann Arbor classification, thus making analysis difficult and statistical significance very much unlikely.

Advanced disease and treatment with chemotherapy (Otten *et al*, 2004) have been elucidated as risk factors for development of TE in patients with solid tumours and also seem to play an a etiologic role in malignant lymphoma (Ottinger *et al*, 1995). Most of the TE we observed occurred during chemotherapy and a higher TE incidence was observed in patients with advanced disease, although statistical analysis failed to reach significance. Thrombosis due to local tumour compression occurred in nine of our TE patients (11%) and was highly associated with advanced-stage disease. Additionally, nine patients (11%) had comorbid solid tumours as another important thrombophilic risk factor (Prandoni *et al*, 1992; Deitcher *et al*, 2003). Activation of coagulation in patients with cancer including malignant lymphoma has been observed in earlier reports, namely increased prothrombin activation, elevation of coagulation factor VIII (F VIII) and impairment of platelet function (Zurborn *et al*, 1986; Nagy and Losonczy, 1987; Nand *et al*, 1987; Falanga *et al*, 1994). There exist no data on inherited or acquired thrombophilia such as anticardiolipin antibodies, antithrombin, protein C or protein S deficiency or the prothrombin gene or factor V Leiden mutation in patients with malignant lymphoma. Zurborn *et al* (1986) found increased levels of F VIII in patients with lung cancer and malignant lymphoma prior to and after application of chemotherapy.

Our study shows that the incidence of TE in patients with malignant lymphoma is comparable to that seen in solid tumours. Since TE may cause substantial morbidity and mortality, oncologists should be alert of this complication. Most thromboembolic complications occur during chemotherapy; thus, prophylactic anticoagulation during this time period should be considered, particularly in patients with hgNHL and additional possible risk factors such as advanced disease.

## Figures and Tables

**Table 1:** Thromboembolic events (TE) in patients with malignant lymphoma: overall and subpopulation analysis

	**Patient number**	**TE patients number**	**TE rate (%)**
All	1038	80	7.70
hgNHL	348	37	10.60
lgNHL	485	28	5.77
HD	193	14	7.25
CNS NHL	12	1	8.33

NHL=non-Hodgkin's lymphoma; hgNHL=high-grade NHL; lgNHL=low-grade NHL; HD=Hodgkin's disease; CNS NHL=central nervous system NHL.

**Table 2:** Thromboembolic event (TE) rate in patients with malignant lymphoma: correlation with histologic subtype

**TE rate hgNHL(%)**	**TE rate lgNHL(%)**	**TE rate HD (%)**	***P*-value**
10.60	5.77		0.012
10.60		7.25	0.222
	5.77	7.25	0.482

See Table 1 footnote for abbreviations.

**Table 3:** Thromboembolic event rate in patients with malignant lymphoma: correlation with gender

	**Male (%)**	**Female (%)**	***P*-value**
All	6.7	9.0	0.198
hgNHL	8.7	12.6	0.297
lgNHL	5.2	6.6	0.555
HD	7.8	6.6	0.788

See Table 1 footnote for abbreviations.

**Table 4:** Thromboembolic event rate in patients with malignant lymphoma: correlation with age

	**⩾60 years (%)**	**<60 years (%)**	***P*-value**
hgNHL	10.7	10.6	1.000
lgNHL	7.1	3.1	0.097
HD	13.3	5.4	0.097

See Table 1 footnote for abbreviations.

**Table 5:** Thromboembolic event rate in patients with malignant lymphoma: correlation with disease stage

	**Stage I and II (%)**	**Stage III and IV (%)**	***P*-value**
hgNHL	8.6	12.6	0.297
lgNHL	—	—	—
HD	7.6	6.8	1.000

See Table 1 footnote for abbreviations.
